# Vitamin D Deficiency and the Presentation of Primary Hyperparathyroidism: A Mini Review

**DOI:** 10.1155/2023/1169249

**Published:** 2023-12-11

**Authors:** Niharika Yedla, Hyon Kim, Anupa Sharma, Xiangbing Wang

**Affiliations:** ^1^Department of Endocrinology, Quincy Medical Group, 1025 Maine Street, Quincy, IL 62301, USA; ^2^Division of Endocrinology, Metabolism and Nutrition, Department of Medicine, Rutgers-Robert Wood Johnson Medical School, MEB 374, 1 RWJ Place, New Brunswick, NJ 08903-0019, USA; ^3^Penn Medicine Princeton Medicine Physicians, 5 Plainsboro Road, Plainsboro, NJ 08536, USA

## Abstract

The clinical presentation of primary hyperparathyroidism (PHPT) has evolved over the years from a symptomatic disorder to a predominantly asymptomatic condition. Altered vitamin D metabolism seems to play a role in the presentation of PHPT and may exacerbate the severity of disease. The epidemiology of PHPT differs in the developing versus the developed world, where more severe phenotypes occur in regions where vitamin D deficiency is common. Although it has been validated that patients with PHPT should be vitamin D sufficient, the threshold to supplement in relation to the severity of PHPT and the degree of vitamin D deficiency remains controversial. This review will highlight some of the controversy regarding vitamin D deficiency and the different phenotypes of PHPT.

## 1. Introduction

Primary hyperparathyroidism (PHPT) is the most common cause of hypercalcemia and is characterized by elevated or inappropriately normal parathyroid hormone (PTH) levels. There are three phenotypes of PHPT [1]. There is classic or symptomatic PHPT which presents with renal or skeletal complications such as nephrolithiasis or osteitis fibrosa cystica. There is asymptomatic PHPT which presents with no obvious signs or symptoms but may still have some renal or skeletal involvement. Also, the newest classification is normocalcemic PHPT (NPHPT) which is defined by normal albumin-corrected or ionized calcium levels with elevated PTH levels. Patients who fit this category may or may not have renal or skeletal complications. Vitamin D is a fat-soluble secosteroid that plays a central role in calcium homeostasis and bone metabolism through the feedback of calcium, phosphate, and PTH. Vitamin D deficiency can cause secondary hyperparathyroidism which must be differentiated from PHPT. The presentation of vitamin D deficiency in PHPT varies geographically. In the Western world, where biochemical screening is common, asymptomatic or NPHPT is more frequently noted. In contrast, symptomatic disease with renal and/or skeletal manifestations is the more predominant phenotype in developing countries [2]. As compared to the general population, vitamin D deficiency is more common in PHPT patients and has been associated with more severe disease [3, 4]. The mechanism behind the association of vitamin D deficiency and PHPT is not clear and remains controversial [5]. In this article, we aim to review the recent research looking into the relationship between the phenotypes of PHPT and 25(OH)D levels as well as vitamin D-binding protein.

### 1.1. Epidemiology and Prevalence of Vitamin D Deficiency in PHPT

Primary hyperparathyroidism and vitamin D deficiency are common conditions, and their effects are inter-related. The operational definition of vitamin D deficiency as given by the Institute of Medicine is a 25(OH)D level ≤20 ng/ml (50 nM/l) [6]. It has also been suggested to define vitamin D insufficiency as 25(OH)D levels between 20 and 30 ng/mL and deficiency as <20 ng/mL [2, 4]. These cutoffs are established for optimal bone health and not in the context of PHPT [4]. The likelihood of 25(OH)D insufficiency (81%) and 25(OH)D deficiency (33%) in PHPT is reported to be much higher than in sex- and age-matched control populations (60% for insufficiency and 20% for deficiency) and remains so with seasonal variations [7, 8]. While 25(OH)D levels are highest in the late summer months (July-August) for people with or without PHPT, the average 25(OH)D level is still overall reduced in PHPT patients [7]. Recent trends show a decrease in the prevalence of vitamin D deficiency in PHPT, possibly from increased supplementation [6, 9]. It is estimated that the prevalence of vitamin D deficiency and insufficiency in PHPT has decreased by 50% and 30%, respectively, with a corresponding decline in PTH levels [10].

### 1.2. Geographic Variation of 25(OH)D Levels in PHPT

With the advent of increased access to population lab screening, developed nations have seen a shift in the clinical presentation of PHPT from a symptomatic disorder to that of a largely asymptomatic condition. In a study comparing PHPT patients in New York to those in Shanghai, the average 25(OH)D levels were found to be significantly lower (13 ng/mL) in the Shanghai cohort versus the New York cohort (36.7 ng/mL, *p* < 0.001) [11]. The patients in the Shanghai cohort also showed biochemical evidence of more severe PHPT with significantly higher PTH (402.1 pg/mL vs. 67.5 pg/mL, *p* < 0.001), alkaline phosphatase (112 U/L vs. 75 U/L, *p* < 0.001), and serum calcium (11.72 mg/dL vs. 10.6 mg/dL, *p* < 0.001) levels compared to the New York cohort. There were no cases of NPHPT in the Shanghai cohort which was partly attributed to the relatively higher prevalence of vitamin D deficiency [11]. Similar trends have been noted in other studies done in China, India, and Brazil, where higher rates of symptomatic disease were noted with lower 25(OH)D levels [12–14]. However, cases of milder or asymptomatic PHPT are rising in developing countries in more recent years [11]. One study in 2021 noted that while the majority (>90%) of PHPT patients in India still present with symptomatic disease, there is a rising prevalence of asymptomatic disease with lower PTH levels and an improved vitamin D status [15].

### 1.3. Vitamin D Deficiency and Classic PHPT

Prior to the ubiquitous screening of calcium in the last several decades, classic PHPT was described as a symptomatic condition with renal and/or skeletal manifestations at the time of diagnosis [9]. Most notably, osteitis fibrosa cystica, which clinically presents as bone pain and is radiographically characterized by a “salt and pepper” appearance of the skull, brown tumors, subperiosteal bone resorption, and osteolytic lesions, is now considered a very rare complication in the developed world [9, 16]. Low bone mineral density (BMD) and fragility fractures are now the more common skeletal signs of symptomatic disease. Classic renal manifestations include hypercalciuria, nephrolithiasis, and nephrocalcinosis.

There have been several studies that suggest vitamin D deficiency (defined as a 25(OH)D level at least <20 ng/mL) is associated with a more severe presentation of PHPT. This is thought to be due to higher PTH levels with higher calcium levels, increased bone turnover markers with lower bone mineral density, and increased parathyroid mass [11, 12, 17–19]. Patients who present with osteitis fibrosa cystica have been shown to have significantly lower 25(OH)D levels than those with asymptomatic disease (16.7 ± 1.1 ng/mL versus 29.9 ± 2.9 ng/mL, *p* < 0.02). They also have significantly higher PTH levels compared to asymptomatic PHPT patients (1352.8 ± 297.2 pg/mL versus 145.0 ± 43.7 pg/mL, *p* < 0.02) [12]. This has also been demonstrated in a cohort of patients with PHPT in India where a majority of patients (90%) present with osteitis fibrosa cystica and have severe vitamin D deficiency with an average vitamin D level of 8.4 ± 5.1 ng/mL [17]. In a study comparing features of PHPT in New York and Shanghai, the patients in China who often presented with symptomatic disease had significantly higher calcium and PTH levels as well as lower vitamin D levels [9]. In an Italian cohort of PHPT patients, those who had 25(OH)D levels below 20 ng/mL were noted to have significantly higher PTH, alkaline phosphatase, and bone turnover markers and significantly lower BMD than patients with 25(OH)D levels above 20 ng/mL [18]. In another Italian cohort of women with sporadic PHPT, it was found that the lowest quartile of 25(OH)D levels correlated with higher PTH levels, higher bone-specific alkaline phosphatase levels, and lower estimated glomerular filtration rates (GFRs). No association was found with serum and urine calcium levels, fracture risk, or nephrolithiasis [20]. Of note, another study similarly showed that a 25(OH)D level less than 20 ng/mL was associated with higher PTH levels but showed no clinical difference in bone mineral density, osteoporosis, fracture, nephrolithiasis, or urinary calcium [10].

### 1.4. Vitamin D Deficiency and Asymptomatic PHPT

In the 1970s, the clinical presentation of PHPT in the Western world shifted from the symptomatic disorder previously described to an asymptomatic condition likely due to widespread biochemical screening. Asymptomatic PHPT is a unique phenotype characterized by mild hypercalcemia without the classic symptoms. Calcium levels are usually less than 1 mg/dL above the upper limit of normal, and the PTH level is less than 2-fold above the upper range of normal [21]. Vitamin D insufficiency or deficiency is commonly noted in asymptomatic PHPT and is associated with higher PTH levels and higher markers of bone turnover such as bone-specific alkaline phosphatase [10, 22]. Low 25(OH)D levels have also been associated with more cortical bone loss and preservation of trabecular bone in these patients [22, 23]. It is, therefore, recommended that 25(OH)D levels be checked in all cases of asymptomatic PHPT and cautious repletion with vitamin D supplementation be initiated if 25(OH)D levels fall below 20 ng/mL [24, 25]. In a study that followed patients with asymptomatic PHPT over the course of 15 years, about a third of patients had progressive disease that eventually met criteria for surgery [26]. Despite the lack of symptoms, patients with seemingly asymptomatic PHPT can still have renal or skeletal involvement which has led to recommendations to continue monitoring asymptomatic disease with radiographic imaging for renal stones, osteoporosis, or fractures [2, 27]. Vitamin D deficiency or insufficiency is less prevalent in asymptomatic PHPT patients due to increased routine vitamin D supplementation [24]. There may also be a role for vitamin D-binding protein (DBP). One study reported significantly lower DBP levels in a cohort of postmenopausal women with PHPT compared to age- and BMI-matched controls. Calculated free 25(OH)D levels and 1,25(OH)_2_D levels were not significantly different between the two groups [28].

### 1.5. Vitamin D Deficiency and Normocalcemic PHPT

In the early 2000s, NPHPT was recognized as a new entity of PHPT with elevated PTH levels in the absence of hypercalcemia [29]. These patients have both normal ionized calcium and total albumin-corrected calcium levels. An increase in PTH measurements as part of the workup for low bone mass and diagnosis of osteoporosis helped discover this biochemical phenotype where calcium levels consistently remain normal [9]. To evaluate this diagnosis, secondary causes of hyperparathyroidism, such as vitamin D deficiency, chronic kidney disease, malabsorption, and medication use such as lithium, diuretics, bisphosphonates, and denosumab, must also be ruled out.

It is possible that slightly elevated PTH levels may be a part of the normal distribution curve in the spectrum of PTH ranges. It is estimated that about 2.5% of the normal population can be a part of this spectrum [9]. Another perspective is that while the increase in PTH may actually cause an increase in the serum calcium level, the concentration may stay within the normal cutoff limits and thus not be detected [30].

There is evidence to suggest that NPHPT patients develop complications of PHPT despite having normal calcium levels and that it does not represent a mild, asymptomatic form. One study concluded that patients seen with NPHPT have more substantial skeletal involvement compared to PHPT and develop more complications in the course of the disease [30, 31]. Another study found higher incidence of kidney stones in NPHPT and similar fracture history in comparison to PHPT. It was hypothesized that NPHPT patients may exhibit resistance to calcium at the bone and kidney levels leading to a higher PTH concentration [32].

The levels of 25(OH)D that would define deficiency in NPHPT are not fully delineated and both >20 ng/mL or >30 ng/mL have been used to rule out deficiency (Table 1). The Institute of Medicine's current cutoff for the diagnosis of vitamin D deficiency is 25(OH)D < 20 ng/mL, which applies to the general population as previously discussed. Some experts suggest repletion to 25(OH)D levels above 30 ng/mL (75 nmol/L), as there is evidence that even vitamin D insufficiency increases PTH levels [9, 33]. Once the 25(OH)D level is in the 30–40 ng/ml range, its previous exponential effect on PTH flattens [37]. Some have even recommended repletion to >40 ng/mL to see the effects on PTH levels [9, 33, 37].

A report that studied free versus total 25(OH)D levels in NPHPT showed that measured free 25(OH)D levels were 20% lower in NPHPT patients than in healthy age-, sex-, and BMI-matched controls (5 vs. 6.2 pg/mL, *p* = 0.013). The total 25(OH)D levels were >30 ng/dL in both the NPHPT and control groups and were not significantly different [35]. This again raised the question of whether total 25(OH)D levels are truly representative of the actual vitamin D status in NPHPT patients and warrants further investigation [37].

### 1.6. Vitamin D Levels in Multiple Endocrine Neoplasia (MEN) Disorders and Familial Hypocalciuric Hypercalcemia (FHH)

It is estimated that about 1–18% of the patients with PHPT have underlying multiple endocrine neoplasia type 1 (MEN1) disease [38]. It presents as a multiglandular entity compared to sporadic PHPT that presents as a single gland adenoma 80–85% of the time. It is associated with recurrent hyperparathyroidism even after presumed successful surgery and the recurrence rates can be up to 50% by 12 years [38–40]. The MEN1 gene product “menin” directly interacts with the vitamin D receptor (VDR) and enhances gene transcription, leading to lower VDR expression in adenoma cells [41]. One study found that 21 out of 31 patients with MEN1 had 25(OH)D levels <10 ng/mL, 9 had levels between 10 and 30 ng/mL, and 1 patient had normal levels, showing the degree of vitamin D deficiency in MEN1 [42]. In addition, PHPT can occur in 20–30% of typical multiple endocrine neoplasia type 2A (MEN2A) syndrome [43].

Familial hypocalciuric hypercalcemia (FHH) is a rare inherited disorder of the calcium-sensing receptor gene, *CASR*. FHH can be biochemically similar to PHPT with an elevated calcium level associated with a normal or high PTH level, a normal 25(OH)D level, and a high 1,25-dihydroxyvitamin D level. The 24-hour urinary calcium excretion, however, is low in FHH. The calculated calcium/creatinine clearance ratio should be <0.01 [9]. It is important for patients to be vitamin D replete for diagnosis as the calcium/creatinine ratio can be <0.01 for patients with PHPT and vitamin D deficiency. Less conclusive studies suggest that vitamin D supplementation in familial PHPT syndromes may show beneficial effects on serum PTH and bone mineral density (BMD); however, more studies are needed [44].

### 1.7. Total or Free 25(OH)D and Vitamin D-Binding Protein in PHPT

Total 25(OH)D can be both free or bound to protein. The vast majority of circulating 25(OH)D is bound to vitamin D-binding protein (DBP) and not biologically available. At present, total 25(OH)D levels are checked to assess the 25(OH)D status and designate deficiency or insufficiency. Most 25(OH)D is bound to DBP, 10–15% is albumin bound, and <1% is unbound in the serum. Different clinical conditions can affect DBP levels, 25(OH)D binding, and total 25(OH)D levels [45]. In a study of 88 patients with PHPT matched with related and unrelated family members without PHPT, it was found that 25(OH)D levels as well as DBP and free and bioavailable (albumin-bound vitamin D plus free vitamin D) 25(OH)D levels were low in PHPT patients. The authors suggested that although low DBP is found in PHPT, it alone cannot be responsible for low vitamin D levels [46]. However, since most of 25(OH)D is DBP bound, lower DBP may cause low total 25(OH)D levels in asymptomatic PHPT patients [47].

Several studies have shown that although total 25(OH)D levels are lower in PHPT compared to controls, the DBP levels are also lower, so the calculated free 25(OH)D levels remain unchanged [28, 47, 48]. Another report showed that calculated free 1,25(OH)_2_D levels are higher in postmenopausal PHPT patients and correlate better with PTH levels in comparison to total 1,25(OH)_2_D [49]. These studies raise the question of whether free 25(OH)D levels may be a better indicator of the vitamin D status in PHPT but this needs further exploration.

### 1.8. Potential Mechanisms of Low 25(OH)D in PHPT

**Figure 1:**
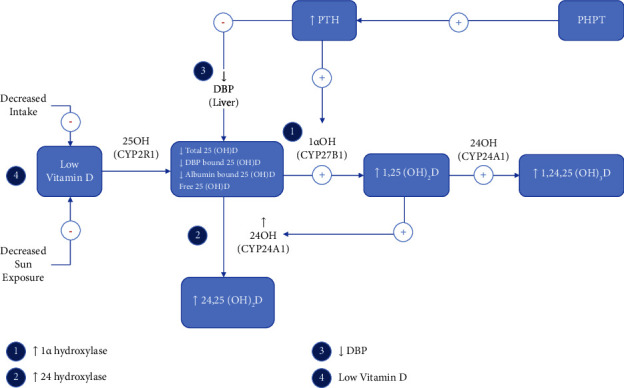
Potential mechanisms of low 25(OH)D levels in PHPT. PHPT: primary hyperparathyroidism; PTH: parathyroid hormone; DBP: vitamin D-binding protein; 25(OH)D: 25-hydroxyvitamin D; 1,25(OH)_2_D: 1,25-dihydroxyvitamin D; 24,25(OH)_2_D: 24,25-dihydroxyvitamin D; 1*α*OH: 1-alpha hydroxylase; 24 OH: 24 hydroxylase.

There is evidence that vitamin D deficiency acts as an inciting factor that worsens the clinical disease status of PHPT [9]. The exact mechanism behind the higher prevalence of low 25(OH)D levels in PHPT is multifactorial, involving low vitamin D intake or production, disturbed 25(OH)D metabolism, and interactions with vitamin D-binding proteins (Figure 1).

PTH enhances the conversion of 25(OH)D to 1,25-dihydroxyvitamin D through increased 1-alpha hydroxylase activity in the kidneys via the CYP27B1 activity. Active vitamin D increases serum calcium via its action on vitamin D receptors (VDRs), which is sensed by the calcium-sensing receptor (CaSR) and feeds back to PTH. Active vitamin D regulates VDR levels in the parathyroid and has an ability to prolong VDR half-life. It also regulates the response of the parathyroid gland to calcium by inducing CaSR gene transcription [50]. Active vitamin D inhibits 1-alpha hydroxylase and activates 24-alpha hydroxylase via the CYP24A1 activity (in the kidneys) to regulate serum calcium concentrations [51]. It can thus be conflicting to have low 25(OH)D levels with normal or even high 1,25-dihydroxyvitamin D levels [9].

The elimination half-life of 25(OH)D is significantly shortened in PHPT through increased inactivation of 25(OH)D by 24-hydroxylation to 24,25(OH)_2_D, its inactive form [51]. This can be accounted for by an increased excretion of inactivated vitamin D derivatives in the feces [4, 51].

In countries such as India, traditional clothing also limits sun exposure and the prevalence of a vegetarian diet adds to nutritional 25(OH)D deficiency [52].

Elevated PTH also decreases DBP production in the liver which in turn leads to decreased total 25(OH)D levels. In a study of 50 PHPT patients, DBP and DBP-bound 25(OH)D levels were found to be lower in PHPT patients compared to healthy controls, while free and bioavailable levels were similar between the groups [47]. DBP and 25(OH)D levels have been noted to revert back to normal after parathyroidectomy [47, 48]. Different gene types of DBP can affect total 25(OH)D levels in PHPT patients [46].

The abovementioned studies provide some insight into the pathophysiology of vitamin D metabolism and lower total 25(OH)D levels in PHPT (Figure 1). They confirm that PHPT is associated with lower total vitamin D levels and that these low levels can affect the biochemical presentation. However, there needs to be more studies to assess clinical endpoints such as fracture risk, incidence of nephrolithiasis, and optimal levels of supplementation [4, 9, 20, 46].

## 2. Supplementation of Vitamin D in PHPT

As previously discussed, low 25(OH)D levels can be associated with a more severe presentation of PHPT with higher PTH and bone turnover marker levels and a larger parathyroid adenoma size than expected [19, 53, 54]. Several studies have tried to analyze the benefits of 25(OH)D supplementation in PHPT with outcomes ranging from hypercalcemia to no significant influence on serum calcium levels or clinical benefits. In a study investigating the effects of vitamin D repletion in patients with mild PHPT for one year, it was noted that mean serum calcium and phosphate levels did not change with the normalization of serum 25(OH)D levels [55]. PTH levels fell by 26% at one year and the serum alkaline phosphatase and urine N-telopeptidelevels were lower than the baseline. Group mean 24-hour urinary calcium excretion showed no changes. This study concluded that 25(OH)D repletion in PHPT does not exacerbate hypercalcemia and could be beneficial by decreasing PTH levels and bone turnover [55]. A meta-analysis of observational studies evaluating the safety of vitamin D replacement in patients with PHPT also showed that vitamin D supplementation can significantly reduce PTH levels without a significant rise in serum or urinary calcium levels [56]. A more recent meta-analysis of over 300 patients with PHPT and vitamin D levels below 30 ng/mL further supported that vitamin D supplementation can significantly decrease PTH and alkaline phosphatase levels without exacerbating hypercalcemia or hypercalciuria [57].

In a randomized clinical trial of PHPT patients who were supplemented with 2800 IU cholecalciferol daily for 52 weeks, an increase in 25(OH)D levels from 23 ng/mL to 38 ng/mL was associated with lower PTH and c-telopeptide levels as well as improvement in the lumbar spine bone mineral density (BMD) without significant changes in serum or urinary calcium [58]. However, in an unblinded study of PHPT patients who were supplemented with calcifediol, 12 out of 27 patients had to stop supplementation due to either hypercalcemia or hypercalciuria [59]. The Fourth International Workshop recommended a threshold 25(OH)D level greater than 20 ng/ml in PHPT [25]. However, the same publication acknowledged the controversy regarding what the optimal cutoff for 25(OH)D should be in the management of PHPT. More recent consensus guidelines, including the Fifth International Workshop, now suggest the repletion of 25(OH)D levels above 30 ng/mL based on the abovementioned studies [1, 60].

We believe that in PHPT with severe hypercalcemia, vitamin D supplementation has to be done with caution to avoid a hypercalcemic crisis. In asymptomatic PHPT patients, vitamin D should be supplemented, while monitoring serum calcium and urinary calcium excretion and with an aim to keep 25(OH)D levels at least between 20 and 30 ng/ml [4, 9, 53]. Vitamin D2 or D3 can be given starting at 1000 IU per day [24]. The repletion of vitamin D to a target level of 40 ng/mL or more may help diagnose NPHPT with more accuracy in select patients. Current guidelines only utilize total 25(OH)D levels, but there have been studies that suggest free 25(OH)D levels may be a better marker for the vitamin D status in NPHPT [35, 37].

### 2.1. Supplementation with Vitamin D before and after Parathyroid Surgery

A recent study compared serum calcium concentrations, PTH levels, calculated free and bioavailable 25(OH)D, and DBP levels before and after parathyroidectomy [48]. The investigators found that serum calcium and PTH levels revert to normal after surgery. DBP as well as DBP-bound 25(OH)D levels increased after surgery likely due to the normalization of PTH. It is important to note that patients in the study received calcitriol after surgery as part of standard postoperative care [48]. In a retrospective cohort study, it was found that vitamin D repletion in PHPT patients undergoing parathyroidectomy decreases hypocalcemia and reduces the length of hospital stay [61].

Vitamin D levels should be measured and supplemented to adequate levels prior to parathyroid surgery [9]. In order to avoid hypocalcemia, we suggest supplementing to a target of 25(OH)D >20 ng/ml for symptomatic patients and to >30 ng/ml for asymptomatic patients. We suggest that the careful repletion of vitamin D to a 25(OH)D level of 40 ng/mL before surgery for NPHPT can be attempted to rule out possible secondary hyperparathyroidism.

## 3. Conclusions

In recent years, the presentation of PHPT has shifted in the Western world to a generally asymptomatic condition with less vitamin D deficiency. Increased rates of screening and supplementation of vitamin D has changed the prevalence of vitamin D deficiency in PHPT and has introduced NPHPT into the clinical spectrum. Vitamin D repletion is recommended in all forms of PHPT, but the threshold to replace to is controversial and should be based on the severity of hyperparathyroidism. Recent studies and meta-analysis suggest that a 25(OH)D threshold level of >30 ng/mL is reasonable to prevent secondary stimulation of PTH secretion and to maintain stable serum and urinary calcium levels. Standard measurements and guidelines only include total 25(OH)D levels, but this may not always be a reliable indicator of the vitamin D status. It may be prudent to also check free 25(OH)D levels along with vitamin D-binding protein levels in the selected patients. Further studies are needed to understand the optimal total 25(OH)D level cutoffs in the different phenotypes of PHPT.

## Figures and Tables

**Table 1:** NPHPT with 25(OH)D levels: age (yo) is expressed as the range or mean ± SD years old; *n* = PHPT patients; *N* = survey subjects; prevalence is expressed as % = *n*/*N*; 25(OH)D cutoff: diagnostic criteria of vitamin D deficiency in the study = expressed as range or mean ± SD ng/dl.

Authors	Age (yo)	n/N Prevalence	% Female	25(OH)D cutoff (ng/mL)	Study population
Lowe et al. [30]	32–78	37/?	95	25D > 20	Referral center USA
				20–54	

Cusano et al. [33]	70 ± 6	9/2364	0	25D > 20	Community based USA
		0.38%		25.2 ± 5	

Chen et al. [34]	60 ± 18.5	11/940	46	25D > 20	Referral center China
		1.12%		26.5 ± 6	

Wang et al. [35]	59.9 ± 5.4	10/940	90	25D > 30	Community based USA
		1.11%		31.9 ± 1.7	

Schini et al. [36]	57–88	11/6280	91	25D > 20	Metabolic bone center UK
		0.18%			

## Data Availability

The data used to support the findings of this study are included within the article.
